# Reduction in chromium sensitization: a retrospective analysis from 2010 to 2024 in a dermatology allergy service^؆^

**DOI:** 10.1016/j.abd.2025.501227

**Published:** 2025-11-06

**Authors:** Mariana de Figueiredo Silva Hafner, Ida Alzira Gomes Duarte, Bruna Cavaleiro de Macedo Souza, Rosana Lazzarini

**Affiliations:** a Dermatology Clinic, Santa Casa de São Paulo, São Paulo, SP, Brazil; b Faculdade de Ciências Médicas, Santa Casa de São Paulo, São Paulo, SP, Brazil

*Dear Editor,*

Among the agents most often involved in allergic contact dermatitis (ACD) are metals, especially chromium (Cr), found in cement, leather, cleaning products, and cosmetics.^1^ Potassium bichromate (PB) is the compound used as a marker of Cr sensitization in patch tests (PT). Previous studies have shown Cr sensitization rates ranging from 12% to 47%, with a downward trend following regulations in European countries.^2^ In Brazil, although specific legislation is still lacking, clinical observations suggest a progressive decrease in cases.

A retrospective study was conducted in a dermatology allergy outpatient clinic between 2010 and 2024, including 1,599 patients with suspected ACD who underwent PT. The battery of tests recommended by the Brazilian Group for Studies on Contact Dermatitis (GBEDC, *Grupo Brasileiro de Estudos em Dermatite de Contato*) was applied to the patients' back. The PT readings were taken at 48 and 96 hours, with the latter being considered for analysis. Patients were grouped by five-year periods: 2010–2014 (group A), 2015–2019 (group B), and 2020–2024 (group C). Sex, occupation, dermatosis location, and positive PT for PB were analyzed, with statistical analysis using the chi-square (χ^2^) test.

Among the 1,599 patients tested, 111 (6.9%) tested positive for PB. The frequency was 11.4% in group A; 5.6% in group B; and 3.6% in group C, with a statistically significant difference between the periods (p < 0.05), indicating a consistent reduction over the years (Table 1). Although most cases occurred in men, no statistically significant difference was observed between the periods in terms of sex ratio, suggesting a uniform reduction between men and women (Table 2).

**Table 1:** Patients tested in the period 2010‒2024 and frequency of positive tests for Potassium Bichromate (PB).

Group	Tested patients	PB (+)	PB (−)
A (2010‒14)	544	62	482
B (2015‒19)	557	31	526
C (2020‒24)	498	18	480
Total	1599	111	1488

χ^2^ = 23.57/p < 0.0001.

**Table 2:** Distribution of patients with positive patch test to potassium bichromate, according to sex. Period 2010‒2024.

Period/Sex	Male	Female	Total
A (2010‒14)	45 (72.5%)	17 (27.5%)	62
B (2015‒19)	20 (64.5%)	11 (35.5%)	31
C (2020‒24)	11 (61%)	7 (39%)	18
Total	76 (68.5%)	35 (31.5%)	111

χ^2^ = 1.283/p > 0.05.

The main dermatitis location were the hands (81% of cases), followed by the upper limbs (42%), trunk and head (16% each), lower limbs (14%), and feet (10%; Table 3). Among the cases with plantar involvement, eight were associated with the use of leather footwear, a material that may contain Cr in its manufacture. The occupation most frequently associated with Cr positivity was mason (46%), followed by homemakers (30.5%), office workers (11%), mechanics, and retirees (4.5% each; Table 4).

**Table 3:** Distribution of contact dermatitis to potassium bichromate according to location. Period 2010‒2024.

Location/Period	UULL^a^	Hands	LLLL^b^	Feet	Trunk	Cephalic segment
A (2010‒2014)	23	48	5	8	7	13
B (2015‒2019)	12	24	6	3	6	5
C (2020‒2024)	11	18	4	0	5	0
Total^c^	46 (42%)	90 (81%)	15 (14%)	11 (10%)	18 (16%)	18 (16%)

a Upper limbs.

b Lower limbs.

c Value greater than the number of patients with positive BP tests, as some patients had more than one location.

**Table 4:** Distribution of patients with positive patch tests to potassium bichromate, according to profession. Period 2010–2024.

Profession/Period	Mason	Homemaker	Mechanic	Office worker	Retired	Other	Total
A (2010‒14)	36	16	4	4	1	1	62
B (2015‒19)	12	12	0	4	2	1	31
C (2020‒24)	3	6	1	4	2	2	18
Total	51	34	5	12	5	4	111

During the analyzed period, 78 masons were treated, with a decreasing distribution over the years (Table 5; p < 0.001). The frequency of positive PB tests among masons also showed a significant reduction (p < 0.006). Of the total number of masons, 27 tested negative for PB (Table 6).

**Table 5:** Number of treated masons. Period 2010‒2024.

Period	Masons	Non-masons	Total
A (2010‒14)	49	495	544
B (2015‒19)	19	538	557
C (2020‒24)	10	488	498
Total	78	1521	1599

χ^2^ = 30.89/p < 0.001.

**Table 6:** Distribution of masons treated in each group and the presence of positive PB tests. Period 2010–2024.

Period	Mason PB+	Mason PB−	Total
A (2010‒14)	36	13	49
B (2015‒19)	12	7	19
C (2020‒24)	3	7	10
Total	51	27	78

χ^2^ = 14.76/p < 0.006.

These findings indicate a progressive reduction in chromium sensitization in the population over the past 15 years. This trend is in line with what has already been observed in European countries after the implementation of restrictions on Cr content in cement and leather, as established by European Directive 2003/53/EC, which limits hexavalent chromium to 2 ppm in cement.^3^, ^4^ In Brazil, although such regulation does not yet exist, a 2009 study detected levels above the European limit in national cement samples.^5^ Despite this, our data suggest a real decrease in sensitization, possibly influenced by factors such as improved working conditions, greater use of Personal Protective Equipment (PPE), replacement of contaminating materials (e.g., refractory bricks used in kilns used in cement manufacturing),^6^, ^7^ and the removal of Cr from cleaning products, detergents, and cosmetics.^8^

The predominance of masons and homemakers among those sensitized reflects direct exposure to Cr present in cement and household products, respectively. The reduction in consultations among masons and the positivity rate in this population suggests that preventive measures may be more effectively implemented, albeit informally, in the construction sector. The anatomical distribution of lesions, particularly on the hands, is consistent with the occupational exposure pattern.^9^, ^10^

In summary, this study shows a consistent reduction in chromium sensitization over the past 15 years, even in the absence of specific national regulations. The observed trend may be the result of a combination of factors, including changes in industrial processes, substitution of materials, and increased awareness among employers and workers about the risks of allergic contact dermatitis. These data reinforce the importance of preventive measures and regulations that limit the presence of allergens in materials for professional and household use.

## ORCID IDs

Ida Alzira Gomes Duarte: 0000-0001-6554-7005; Bruna Cavaleiro de Macedo Souza: 0000-0002-3639-9995; Rosana Lazzarini: 0000-0002-4893-3593

## Financial support

None declared.

## Authors’ contributions

Mariana de Figueiredo Silva Hafner: Conception and planning of the study; elaboration and writing of the manuscript; obtaining, analyzing, and interpreting the data; effective participation in research orientation; intellectual participation in propaedeutic and/or therapeutic conduct of studied cases; critical review of the literature; critical review of the manuscript; approval of the final version of the manuscript.

Ida Duarte: Conception and planning of the study; elaboration and writing of the manuscript; obtaining, analyzing, and interpreting the data; effective participation in research orientation; intellectual participation in propaedeutic and/or therapeutic conduct of studied cases; critical review of the literature; critical review of the manuscript; approval of the final version of the manuscript.

Bruna Cavaleiro de Macedo Souza: Obtaining, analyzing, and interpreting the data; approval of the final version of the manuscript.

Rosana Lazzarini: Critical review of the literature; critical review of the manuscript; approval of the final version of the manuscript.

## Availability of research data

The entire dataset supporting the results of this study was published in the article itself.

## Conflicts of interest

None declared.
